# Supplementary material for: IPSC-Derived Neuronal Cultures Carrying the Alzheimer’s Disease Associated TREM2 R47H Variant Enables the Construction of an Aβ-Induced Gene Regulatory Network
Source: Int J Mol Sci. 2020 Jun 25;21(12):4516. doi: 10.3390/ijms21124516 (PMC7350255; doi:10.3390/ijms21124516)
Supplement: Supplementary file 1 [file ijms-21-04516-s001.zip › Supplementary files/Table S3.pdf]

Supplementary table 3

Gene name	KEGG orthology	Ratio CON8_Aβ/ CON8_CTR	Ratio TREM2_Aβ/ TREM2_CTR
HLA-DMA	MHCII	1.33	N.S.
HLA-DMB	MHCII	1.58	N.S.
HLA-DOA	MHCII	1.37	N.S.
HLA-DPB1	MHCII	1.38	N.S.
HLA-DQA1	MHCII	1.46	N.S.
HLA-DQB1	MHCII	1.57	N.S.
HLA-DRB1	MHCII	1.34	N.S.
HLA-F	MHCI	1.60	N.S.
TUBB4A	TUBA	N.S.	0.73
TUBB4B	TUBB	N.S.	0.73
DYNC1H1	Dynein	N.S.	0.70
LAMP2	LAMP	N.S.	0.74
ATP6V1A	vATPase	N.S.	0.70
ACTB	F-actin	N.S.	0.69
THBS1	TSP	N.S.	0.71
CALR	CALR	N.S.	0.72
TUBA1C	TUBA	N.S.	0.67
