# Supplementary material for: IPSC-Derived Neuronal Cultures Carrying the Alzheimer’s Disease Associated TREM2 R47H Variant Enables the Construction of an Aβ-Induced Gene Regulatory Network
Source: Int J Mol Sci. 2020 Jun 25;21(12):4516. doi: 10.3390/ijms21124516 (PMC7350255; doi:10.3390/ijms21124516)
Supplement: Supplementary file 1 [file ijms-21-04516-s001.zip › Supplementary files/Table S5.pdf]

Supplementary table 5

Gene name	KEGG orthology	Ratio CON8_Aβ/ CON8_CTR	Ratio TREM2_Aβ/ TREM2_CTR
XBP1	XBP	N.S.	3.02
HERPUD1	HERP	N.S.	2.47
DDIT3	CHOP	1.35	2.12
ERP29	PDIs	N.S.	1.71
ATF4	ATF4	N.S.	1.44
SSR1	TRAP	N.S.	1.37
SEC63	Sec62/63	N.S.	1.36
RAD23A	RAD23	N.S.	1.36
P4HB	PDIs	N.S.	0.67
CALR	CRT	N.S.	0.72
HSP90B1	GPR94	N.S.	0.73
DNAJC3	Hsp40	N.S.	0.74
HSPH1	NEF	N.S.	0.74
CAPN1	Calpain	N.S.	0.75
HSPA5	BiP	N.S.	0.75
