# Supplementary material for: IPSC-Derived Neuronal Cultures Carrying the Alzheimer’s Disease Associated TREM2 R47H Variant Enables the Construction of an Aβ-Induced Gene Regulatory Network
Source: Int J Mol Sci. 2020 Jun 25;21(12):4516. doi: 10.3390/ijms21124516 (PMC7350255; doi:10.3390/ijms21124516)
Supplement: Supplementary file 1 [file ijms-21-04516-s001.zip › Supplementary files/Table S6.pdf]

Supplementary table 6

Gene	5'-3' Forward	5'-3' Reverse	Template [bp]
PV	AAAGAGTGCGGATGATGTGAAG	ACCCCAATTTTGCCGTCCC	186
SOM	GCTGCTGTCTGAACCCAAC	CGTTCTCGGGGTGCCATAG	138
CALB2	GCTCCAGGAATACACCCAAA	CAGCTCATGCTCGTCAATGT	208
GAD67	AGGCAATCCTCCAAGAACC	TGAAAGTCCAGCACCTTGG	124
GAD65	CGCATGGTCATCTCAAACC	AGTGGAACAGCTTGGTGAGC	114
TREM2	TCGAGGATGCCCATGTGGAG	TTAGGAAAGACCCATCGCTGT	146
RPS16	GCTATCCGTCAGTCCATCTCCAA	CCTTCTTGGAAGCCTCATCCAC	73
