# Supplementary figures and images for: IPSC-Derived Neuronal Cultures Carrying the Alzheimer’s Disease Associated TREM2 R47H Variant Enables the Construction of an Aβ-Induced Gene Regulatory Network
Source: Int J Mol Sci. 2020 Jun 25;21(12):4516. doi: 10.3390/ijms21124516 (PMC7350255; doi:10.3390/ijms21124516)

S1 Fig

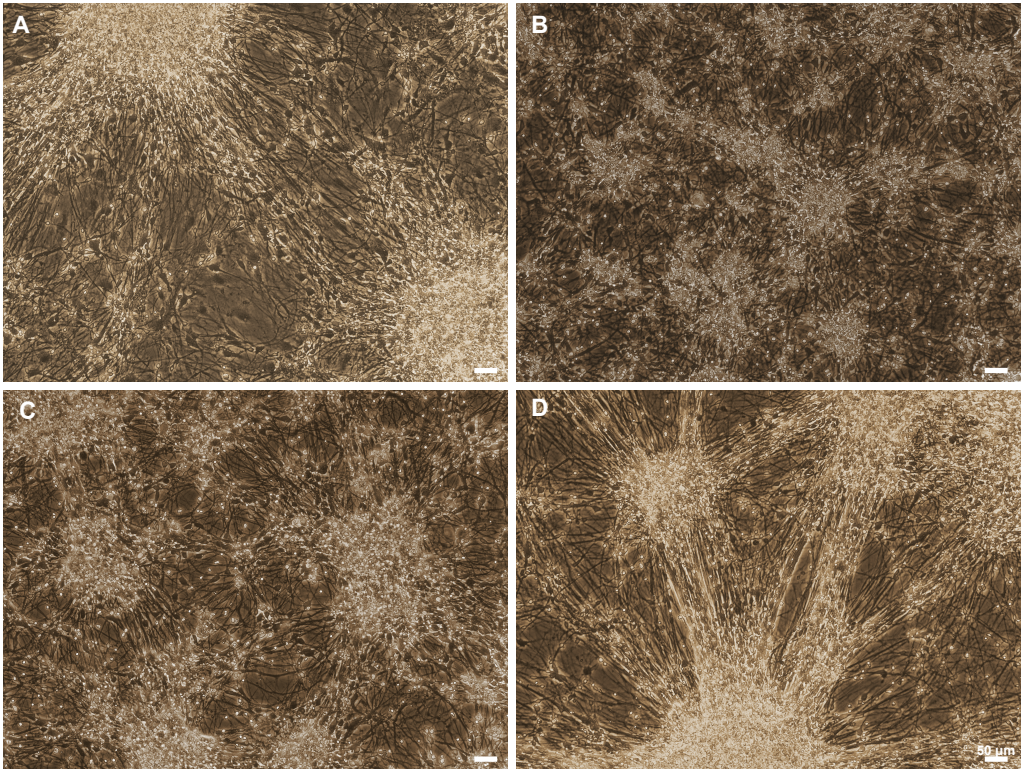

E

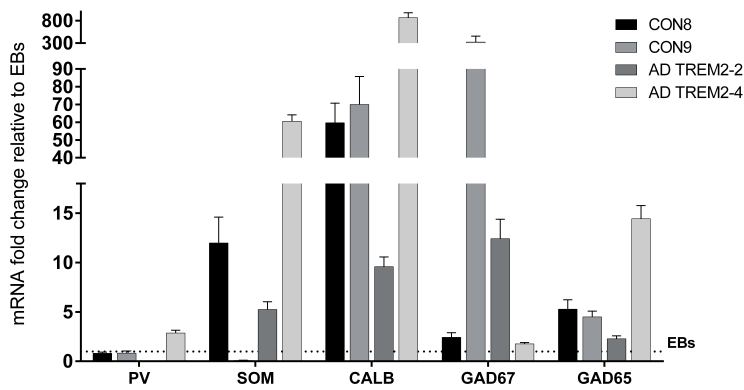

Supplement: Supplementary file 1 [file ijms-21-04516-s001.zip › Supplementary files/Figure S1.pdf]
